# *Zbtb16* (PLZF) is stably suppressed and not inducible in non-innate T cells via T cell receptor-mediated signaling

**DOI:** 10.1038/srep12113

**Published:** 2015-07-16

**Authors:** Sai Zhang, Amale Laouar, Lisa K. Denzin, Derek B. Sant’Angelo

**Affiliations:** 1 Graduate School of Biomedical Sciences, Rutgers Robert Wood Johnson Medical School, New Brunswick, NJ 08901, USA; 2 Department of Surgery, Rutgers Robert Wood Johnson Medical School, New Brunswick, NJ 08901, USA; 3 Department of Pediatrics, Rutgers Robert Wood Johnson Medical School, New Brunswick, NJ 08901, USA; 4 Child Health Institute of New Jersey, Rutgers Robert Wood Johnson Medical School, New Brunswick, NJ 08901, USA

## Abstract

The transcription factor PLZF (promyelocytic leukemia zinc finger; *zbtb16*) is essential for nearly all of the unique characteristics of NKT cells including their rapid and potent response to antigen. In the immune system, zbtb16 expression is only found in innate cells. Conventional T cells that ectopically express PLZF spontaneously acquire an activated, effector phenotype. Activation induced expression of lineage defining transcription factors such as T-bet, FoxP3, RORγt, GATA3 and others is essential for naïve T cell differentiation into effector T cells. In this study, we used sensitive genetic-based approaches to assess the induction of PLZF expression in non-innate T cells by T cell receptor (TCR)-mediated activation. Surprisingly, we found that PLZF was stably repressed in non-innate T cells and that TCR-mediated signaling was not sufficient to induce PLZF in conventional T cells. The inactivated state of PLZF was stably maintained in mature T cells, even under inflammatory conditions imposed by bacterial infection. Collectively, our data show that, in contrast to multiple recent reports, PLZF expression is highly specific to innate T cells and cannot be induced in conventional T cells via TCR-mediated activation or inflammatory challenge.

The specific expression of various members of BTB-ZF family of transcription factors is now recognized to be the lineage-defining event for multiple steps in immune system development and/or function. Examples include ThPOK (*zbtb7B*), which is required for CD4 T cell differentiation, LRF (*zbtb7A*), which is required for the B cell versus T cell lineage decision, *zbtb1*, which is required for entry of precursor cells into the lymphoid lineage and Bcl6 (*zbtb27*), which is essential for the development of both T follicular helper cells and germinal center B cells^1^. Furthermore, in general, ectopic expression of these transcription factors dramatically alters the function and phenotype of lymphocytes. For example, ectopic expression of ThPOK in CD8 T cells causes the loss of CD8 expression, the acquisition of CD4 expression and the induction of multiple helper cell functionalities^2^. Lineage appropriate expression of BTB-ZF genes is, therefore, essential for proper immune system function.

The transcription factor promyelocytic leukemia zinc finger (PLZF), encoded by *zbtb16*, is essential for the development and function of invariant^3^,^4^ and polyclonal natural killer T (NKT) cells^5^, Vγ1.1Vδ6.3 γδ NKT cells^6^ and innate lymphoid cells^7^ in mice. In humans, in addition to invariant NKT cells, PLZF also appears to be important for the development and function of mucosal-associated invariant T (MAIT) cells^8^,^9^, NK cells and γδ T cells^9^. Innate T cells are distinct from other T cells in that they secrete copious amounts of multiple cytokines minutes after primary activation^5^,^10^,^11^. This rapid response can help effectively clear pathogens, but may also contribute to allergy and autoimmunity^12^−^14^. In the absence of PLZF, NKT cells fail to release cytokines upon primary stimulation and, more similar to conventional T cells, require secondary stimulation to fully elaborate their effector functions^3^,^4^. Ectopic expression of PLZF in conventional T cells, on the other hand, results in the spontaneous acquisition of an activated phenotype and the capacity to secrete effector cytokines such as IFN-γ, GM-CSF and IL-17, upon primary activation^15^,^16^,^17^.

Although it is clear that the early growth response proteins Egr1 and Egr2^18^, the inhibitor of DNA binding proteins Id2 and Id3^19^,^20^,^21^ and the E proteins HEB and E2A^22^ are important for PLZF expression, NKT cell specific factors that control expression have not yet been defined. Recent studies indicate that strong TCR mediated signaling can induce PLZF expression in non-innate T cells^18^,^23^,^24^. These data are consistent with the idea that NKT cell precursors receive an agonist TCR signal during their development in the thymus, which is important for directing them into innate T cell lineage^10^,^25^. Here, we used sensitive, genetic-based PLZF-reporter systems to examine PLZF induction *in vitro* and *in vivo* in mature T cells, developing thymocytes, as well as in effector and memory T cells that had responded to bacterial infection. We found that under multiple different experimental conditions, PLZF expression was not inducible in non-innate lymphocytes. Consistent with data showing epigenetic silencing of the *zbtb16* gene in naïve and effector T cells, we conclude that *zbtb16* is inactivated and stably suppressed in conventional T cells. PLZF expression is, therefore, highly specific to the innate lineage and cannot be induced as a consequence of activation.

## Results

### TCR-mediated signaling is not sufficient to induce PLZF in mature T cells

To directly monitor the expression of PLZF in live cells, we utilized **P**LZF-**eG**FP (PEG) reporter mice. Modest changes in PLZF expression have substantial consequences on NKT cell function^17^ and, therefore, we were reluctant to directly mutate the *zbtb16* locus. Rather, we chose to modify a ~ 232 kb bacteria artificial chromosome (BAC) that spans the entire *zbtb16* gene, including more than 20kb 5' and 3' of the gene. eGFP was inserted in-frame with the natural start codon for *zbtb16*. The eGFP gene included a stop codon so PLZF itself is not expressed from the BAC. As we previously published, expression of eGFP precisely mimics PLZF expression^26^,^27^. For example, FACS analysis showed that eGFP expression was nearly identical to the highly regulated expression of PLZF that occurs during the development of NKT cells in the thymus (Supplementary Fig. 1a). Also, GFP was not detected in conventional CD4 or CD8 T cells (Supplementary Fig. 1b). Moreover, intra-nuclear staining of sorted GFP positive and GFP negative cells with an anti-PLZF monoclonal Ab showed that the GFP positive cells expressed PLZF, whereas GFP negative cells did not^26^.

The advantages of using PEG mice to study PLZF expression are considerable. The analysis of nuclear resident proteins by FACS often results in non-specific antibody staining, which is a particular problem with all of the anti-PLZF antibodies. For the studies reported here, on the other hand, direct analysis of live cells was done, which enabled highly accurate exclusion of dead cells by the use of the DNA specific stain, DAPI. Furthermore, the use of the GFP reporter also allowed for functional tests of sorted cell populations.

**Figure 1:**
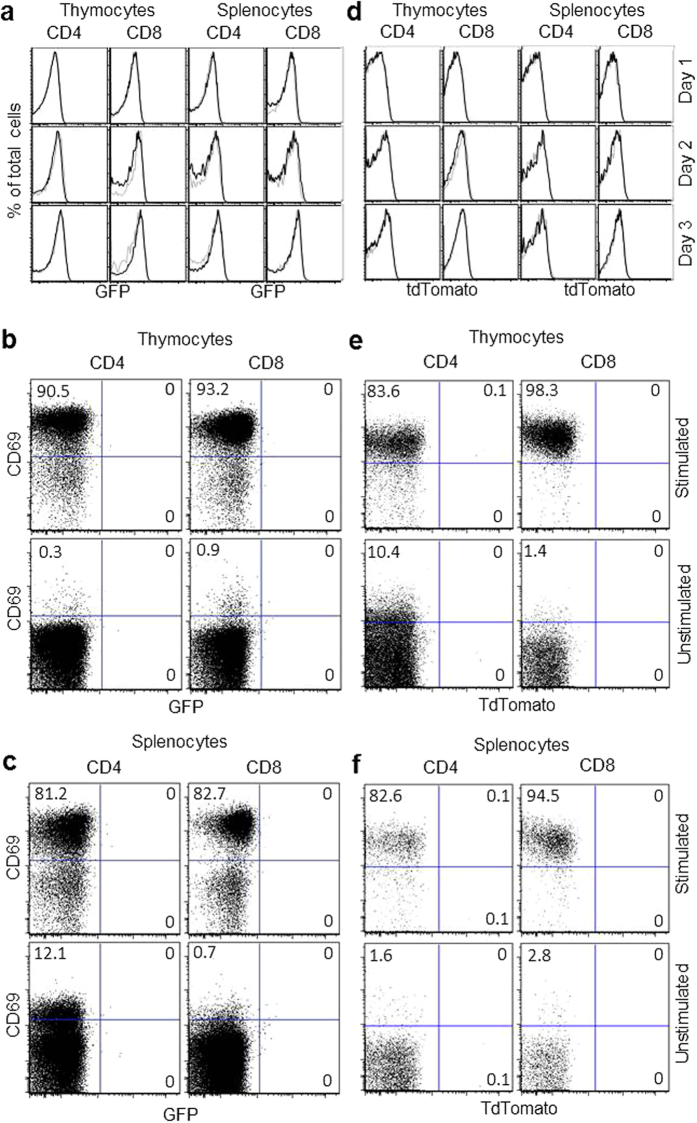
PLZF is stably suppressed in conventional T cells following strong, *in vitro* TCR mediated activation. (**a**) CD4SP and CD8SP T cells were sorted from the thymuses and spleens of WT (grey line) and PEG (black line) mice followed by stimulation with anti-CD3/anti-CD28. After 3 days in culture, the T cells were analyzed for GFP expression by FACS. Live (DAPI negative) T cells are shown. (**b**) and (**c**) T cells shown in (**a**) were also stained for CD69 to show that subpopulations of GFP expressing cells were not detected among the activated T cells. Unstimulated cells were cultured without antibodies. (**d**) Similar to experiments described in (**a**), spleen T cells and thymocytes from Pcre x R26T mice were collected, activated and analyzed by FACS. (**e**) and (**f**) show CD69 expression on cells cultured with and without antibodies. Numbers in dot plots show the percentage of events in each quadrant. Representative FACS plots from 1 of 3 independent experiments are shown.

The PEG mice were used to test for PLZF expression in conventional T cells and thymocytes following activation. This was done by sorting CD4 and CD8 single positive (SP) thymocytes and CD4^+^ and CD8^+^ spleen T cells from both wild type and PEG mice. Cells actively expressing PLZF (GFP positive) were excluded during the sort. The purified T cells were cultured with soluble anti-CD28 with or without plate-bound anti-CD3 and analyzed by FACS at time points up to three days post stimulation. GFP expression was not detected in either thymocytes or splenic T cells at these time points (Fig. 1a). Multicolor analysis showed that small populations of GFP expressing cells were not detectable, even among the CD69^hi^ cells, which clearly had been fully activated (Fig. 1b,c).

These data show that sustained expression of PLZF cannot be induced by activation. However, it is possible that the transcription factor is transiently expressed. To test this possibility, we carried out “fate-mapping” experiments that would definitively detect even brief or low levels of expression of PLZF. Utilizing the same approach that was used for the PEG mice, we generated BAC transgenic mice that express the Cre recombinase in all PLZF expressing cells. The PLZF-Cre (PCre) mice were then crossed with *Rosa 26*-tdTomato (R26T) mice, in which the Rosa 26 locus was modified with a tdTomato fluorescent gene that is preceded by a loxP flanked STOP cassette. Removal of the STOP cassette by Cre recombinase, in this case specifically in PLZF-expressing cells, results in permanently labeled tdTomato positive cells, even if the cells extinguish expression of PLZF at a later time.

FACS analysis showed that, as expected, CD1d tetramer positive NKT cells uniformly expressed tdTomato (Supplementary Fig. 1c). Interestingly, a proportion of non-PLZF expressing conventional T cells were also found to be positive for tdTomato (Supplementary Fig. 1d). Since PLZF is not expressed in resting, naïve conventional T cells, this finding indicated that PLZF was induced during an earlier stage of development that has yet to be clearly defined. These data are nearly identical to data in a recent report using a different PLZF-Cre system^7^.

TdTomato negative CD4SP and CD8SP thymocytes and CD4^+^ and CD8^+^ spleen T cells were purified by cell sorting and activated *in vitro*, as described above. No tdTomato expressing cells were detected at any time point among any of the tested cell populations (Fig. 1d). Even among the fully activated CD69^hi^ cells, it is clear that no tdTomato positive cells can be detected (Fig. 1e,f). Combined these data clearly show that PLZF cannot be induced, even transiently, in thymocytes or T cells by stimulation through the TCR.

### Stable inactivation of PLZF is maintained following *in vivo* activation

**Figure 2:**
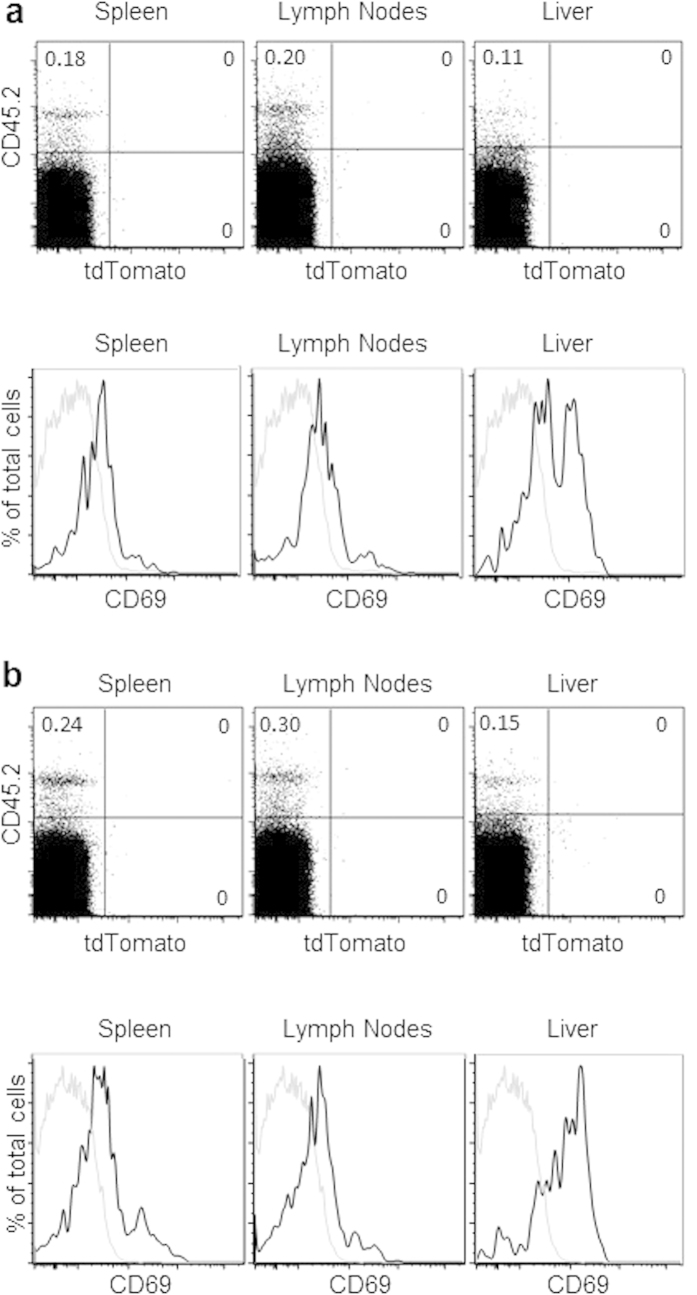
PLZF expression is not induced following TCR mediated activation *in vivo*. TdTomato-negative T cells were sorted from the spleen (**a**) and the thymus (**b**) from PCre x R26T mice and adoptively transferred into B6.SJL mice followed by i.p. injection of 50 μg of anti-CD3. Two weeks later, cells from the spleen, lymph nodes and liver of the recipient mice were analyzed. Transferred cells were identified by the congenic maker CD45.2 and analyzed for the expression of TdTomato by FACS. CD69 expression indicates the cells were activated by the antibodies (black line showing mice with injection and grey line showing spleen cells in control mice with no activation). Cells were gated on the DAPI^−^CD3+ population. Numbers in dot plots show the percentage of events in each quadrant. Representative FACS plots from 1 of 3 independent experiments are shown.

*In vitro* activation of lymphocytes clearly has limitations that might prevent induction of PLZF. Therefore, we next established a cell transfer system following by *in vivo* activation. Two million purified tdTomato negative conventional spleen T cells were adoptively transferred by intraperitoneal injection into unmanipulated B6.SJL mice. T cell activation was induced by injecting the mice with 50 μgs of anti-CD3 antibody. Two weeks later, the mice were sacrificed and lymphocytes were analyzed by FACS. The transferred cells were identified by the expression of the congenic marker CD45.2^+^, which is not expressed by the host B6.SJL mice. Transferred T cells were identified in the spleen, lymph node and livers of the mice (Fig. 2a). CD69 staining indicated that the cells were activated. None of the transferred T cells expressed tdTomato, showing that PLZF had not been expressed at any time point following activation (Fig. 2a).

Next, we adoptively transferred total tdTomato negative thymocytes. Two weeks following activation with anti-CD3, cells were harvested and tested for expression of tdTomato. Once again, it was clear that PLZF had not been induced in activated cells since none of the transferred thymocytes were tdTomato positive (Fig. 2b). Therefore, *in vivo* activation of non-innate T cells and thymocytes does not induce PLZF expression.

### PLZF is not induced in developing thymocytes as a consequence of SLAM family member signaling

SAP (SLAM associated protein) deficient mice have a near complete loss of NKT cells, demonstrating the requirement for the SLAM (signaling lymphocytic activation molecule) family receptors for development and expansion of NKT cells^28^. It has also been shown that homotypic interactions between Slamf1 and Slamf6 are essential for the complete maturation of NKT cells^29^. Importantly, SAP is not necessary for PLZF expression^3^,^29^. SAP is also not required for the acquisition of innate-like effector functions in T cells ectopically expressing PLZF^15^. Nonetheless, it is still reasonable to propose that this signaling pathway plays a role in the induction of PLZF in lymphocytes. Of particular note, recent data showed that TCR signaling combined with SLAM signaling induced the expression of PLZF in nearly all pre-selection-DP (PS-DP) thymocytes^23^.

**Figure 3:**
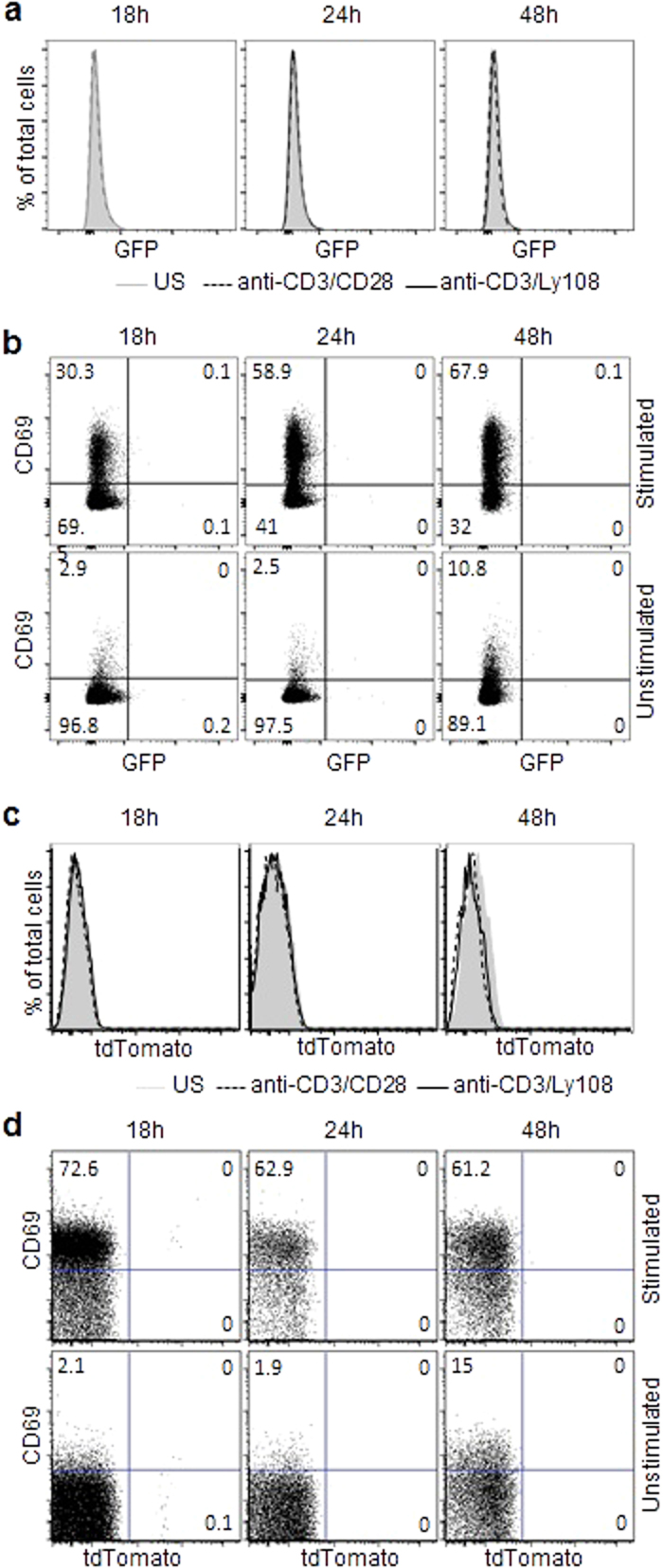
TCR mediated activation combined with costimulation via Ly108 does not induce PLZF expression. PS-DP thymocytes were sorted from PEG (**a**) or PCre x R26T (**c**) mice, stimulated with anti-CD3/anti-CD28 or anti-Ly108 for 48 hours, then analyzed by FACS. GFP and tdTomato expression in live T cells is shown. US, unstimulated. (**b** and **d**), Sorted PS-DP thymocytes, as in (**a**) and (**c**), were stained for surface expression of CD69 after stimulation. Numbers in dot plots show the percentage of events in each quadrant. Representative FACS plots from 1 of 3 independent experiments are shown.

To examine the role of SLAM signaling in PLZF induction, we sorted GFP-negative preselection double positive (PS-DP) thymocytes (CD3^lo^CD25^−^CD44^−^) from PEG mice. The cells were then stimulated, *in vitro*, with anti-CD3 and anti-CD28 or with anti-CD3 and an antibody against the SLAM family member Ly108 (SLAMF6) for up to 48 hours. Since intra-nuclear staining for PLZF was not required, we were able to accurately separate live cells from dead using the DNA specific stain, DAPI. This was important since immature thymocytes undergo extensive cell death *in vitro*. We found that PLZF expression was not induced in PS-DP thymocytes (Fig. 3a). Upregulation of CD69 clearly indicated that the cells have been fully activated (Fig. 3b). Similarly, we sorted PS-DP thymocytes from PCre x R26T mice and performed the same experiment. Results showed that PLZF was not even transiently expressed in these cells after stimulation (Fig. 3c,d).

To further confirm these results, we sorted PS-DP thymocytes from WT mice, activated as described above, followed by staining with a monoclonal antibody against PLZF. To circumvent the background staining introduced by cell death, the LIVE/DEAD Fixable Dead Cell Stain Kit (Invitrogen) was used to exclude dead cells. Results showed no induction of PLZF protein in PS-DP thymocytes (Supplementary Fig. 2a,b). NKT cells were also stained as a positive control for PLZF expression (Supplementary Fig. 2a).

**Figure 4:**
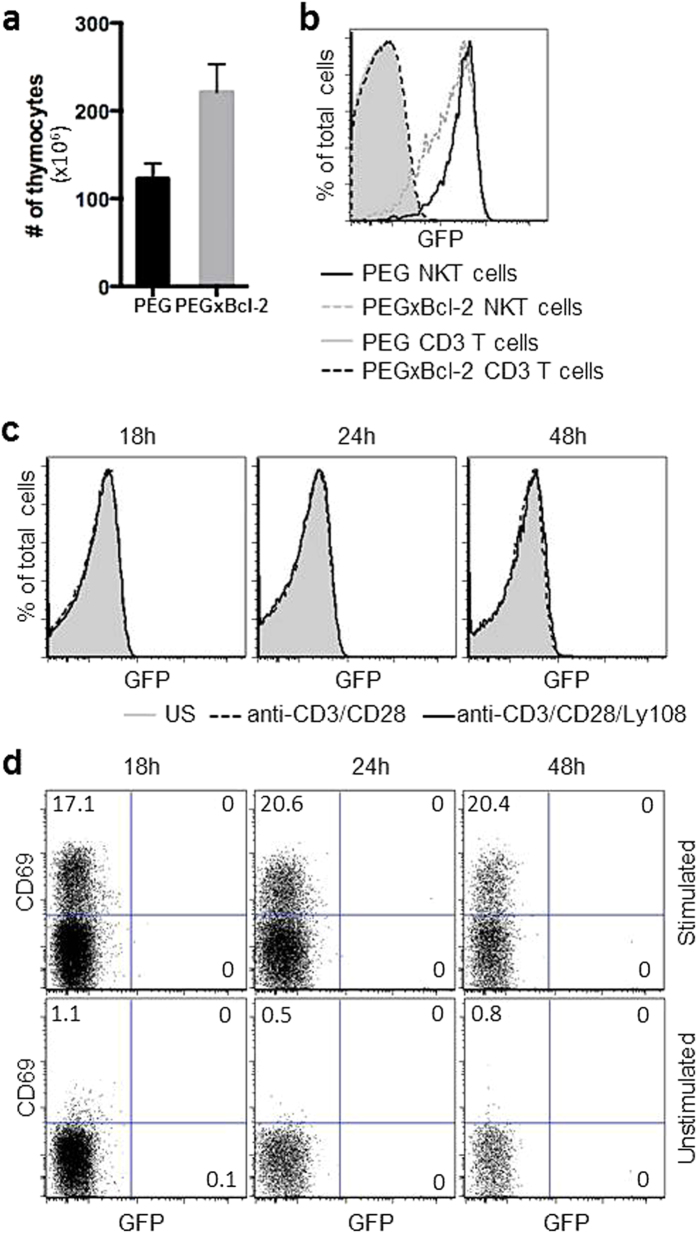
PLZF is not induced in PS-DP thymocytes in PEG × Bcl-2 mice. (**a**) Total number of thymocytes for PEG and PEG × Bcl-2 mice. (**b**) GFP expression profile of NKT cells and CD3+ T cells from PEG and PEG × Bcl-2 mice. (**c**) PS-DP thymocytes were sorted from PEG × Bcl-2 mice, stimulated with anti-CD3/anti-CD28 or anti-Ly108 for 48 hours, then analyzed by FACS. GFP expression in live T cells is shown. (**d**) Sorted were also stained for surface expression of CD69 after stimulation. Numbers in dot plots show the percentage of events in each quadrant. Representative FACS plots from 1 of 3 independent experiments are shown.

Finally, to sustain viable DP thymocytes in culture for a longer time, we crossed PEG mice to mice carrying a H2K-Bcl-2 transgene that forces expression of the anti-apoptotic factor, Bcl-2, in all nucleated cells^30^. As previously reported^31^, the expression of Bcl-2 from this transgene resulted in increased numbers of thymocytes (Fig. 4a). Expression of eGFP in PEG × Bcl-2 mice, however, was similar to control PEG mice (Fig. 4b). Sorted GFP negative, PS-DP thymocytes from PEG × Bcl-2 mice were cultured with anti-CD3 and anti-CD28 or anti-CD3 and anti-Ly108 antibodies. Again, however, there was no detectable induction of PLZF expression, even among the CD69^hi^ cells (Fig. 4c,d). Together, these data showed that PLZF could not induced in immature, developing thymocytes by activation of the SLAM signaling pathway.

### PLZF expression is suppressed during thymocyte development *in vivo*.

Although data are indirect, there is a general consensus that developing NKT cells receive unusually strong “agonist” TCR mediated signaling during development in the thymus. This strong signaling is considered to be a key mediator for the direction of thymocytes into the innate T cell lineage^18^,^32^,^33^,^34^,^35^. For example, it was recently shown that NKT cells have somewhat higher levels of the broadly expressed transcription factors, Egr1 and Egr2^18^. In T cells, these factors can be further induced via the TCR and, therefore, higher levels indicate stronger signaling. Furthermore, Egr2 was shown to bind the promoter of PLZF and, therefore, potentially induces PLZF transcription. This interpretation was directly supported by data showing that PLZF could be induced in thymocytes by injection of mice with anti-TCR antibodies, which caused strong TCR signaling^18^.

**Figure 5:**
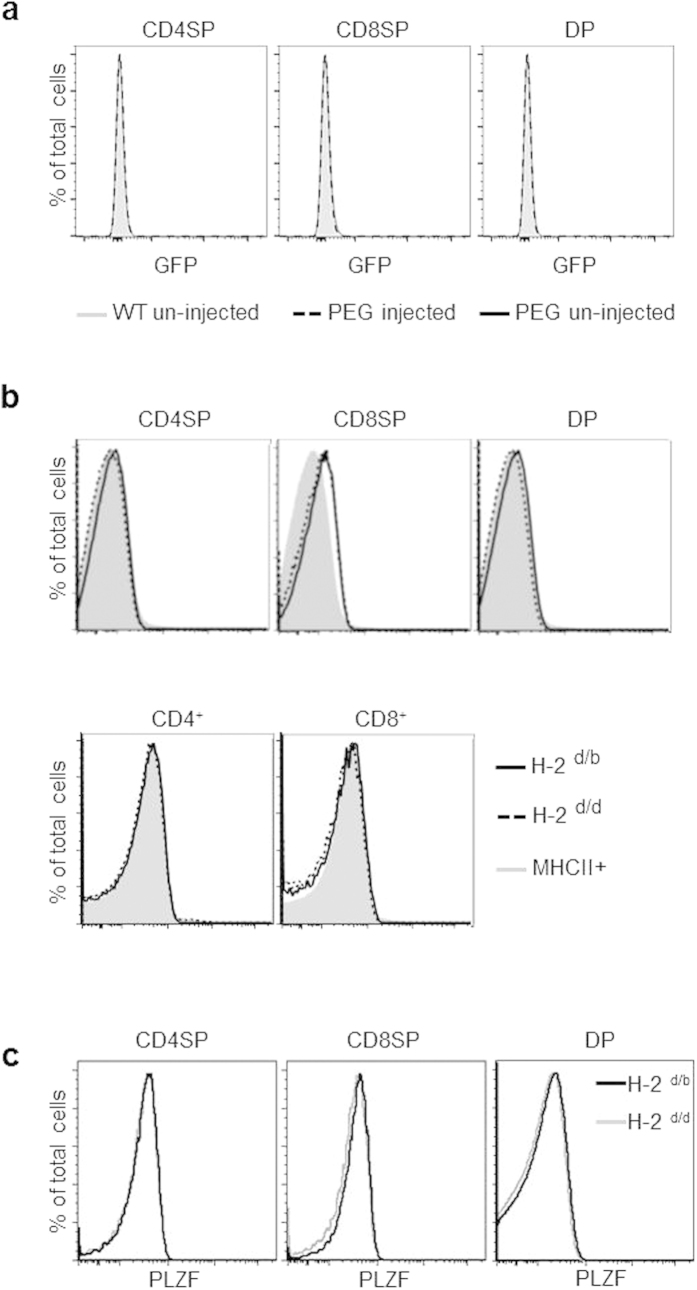
PLZF induction is suppressed during thymocyte development. (**a**) Mice were i.v. injected with 50 μg of anti-CD3. 30 hours later, GFP expression in CD4SP, CD8SP and DP thymocytes from injected and un-injected PEG mice plus un-injected WT mice were examined by FACS. (**b**) GFP expression profiles of thymocytes and spleen T cells from PEG × DO11.10 mice with H-2^d/d^ and H-2^d/b^ haplotypes. Cells were gated on the DAPI^−^CD1d-tetramer^−^γδTCR^−^ population. Indicated populations from the thymus or spleen are shown in histograms. MHCII^+^ cells, which do not express PLZF, were used as negative controls for PEG expression. Representative FACS plots from 1 of 3 independent experiments are shown. (**c**) CD4SP, CD8SP and DP thymocytes from the indicated DO11.10 mice were made permeable and stained for intracellular PLZF expression.

We explored the ability of PLZF to be induced in non-innate thymocytes during development first by taking advantage of our GFP reporter mice to re-examine the possibility that a short, agonist signal delivered by antibodies against the TCR would induce PLZF. PEG mice were intravenously injected with 50 μgs of anti-CD3. 30 hours later, the mice were euthanized and thymocytes were analyzed for upregulation of eGFP. Consistent with this type of agonist activation, total thymocyte cell numbers were reduced by more than 75% (data not shown). There was, however, clearly no upregulation of PLZF in thymocytes following agonist signaling (Fig. 5a). It should be noted that there was a reproducible increase in the percentage of both NKT and γδ T cells (Supplementary Fig. 3). As previously published^6^, approximately 15% of the γδ T cells expressed PLZF (data not shown). This enrichment for NKT and γδ T cells likely explains the increase in PLZF expression that was previously reported following similar activation of thymocytes^18^.

In addition to TCR signaling, however, other undefined *in vivo* signals are potentially required to induce PLZF expression. Therefore, we next established a system in which developing thymocytes would receive different strengths of TCR mediated *in vivo* signaling via interactions with self-peptide:self-MHC. To accomplish this, we utilized mice carrying transgenes for the MHC class II restricted TCR, DO11.10^36^. Thymocytes expressing the DO11.10 TCR are positively selected in BALB/c mice as a result of productive interactions with the MHC class II allele, I-A^d^ 36. The DO11.10 TCR also functionally interacts with the I-A^b^ allele. This interaction is stronger, however, and results in partial negative selection of D011.10 expressing thymocytes^37^. The strength of the signal delivered to DO11.10 thymocytes, therefore, can be modulated by changing the expressed MHC allele. This was done by breeding DO11.10, I-A^d/d^ mice, to C57BL/6, I-A^b/b^ mice, to generate heterozygous I-A^d/b^ mice. To increase the sensitivity of PLZF detection, we also introduced the PLZF-eGFP reporter into the system.

The introduction of the I-A^b^ MHC allele, which presents a high avidity ligand for the DO11 TCR, produced the reported phenotype of increased number of TCR expressing double negative (CD4-CD8-) cells and an overall decrease in cellularity (~30–50% fewer cells, data not shown). Both of these characteristics are a consequence of increased strength of TCR mediated signaling. FACS analysis, however, clearly showed that there was no detectable PLZF expression in CD4SP, CD8SP, DP thymocytes or in spleen T cells as a consequence of this strong signal during development (Fig. 5b). Results were further confirmed by direct staining for the PLZF protein (Fig. 5c). Endogenously provided strong TCR signals during thymocyte development, therefore, cannot induce PLZF in conventional T cells.

### Type 1 inflammation does not induce PLZF during the effector T cell response or during the memory T cell response

**Figure 6:**
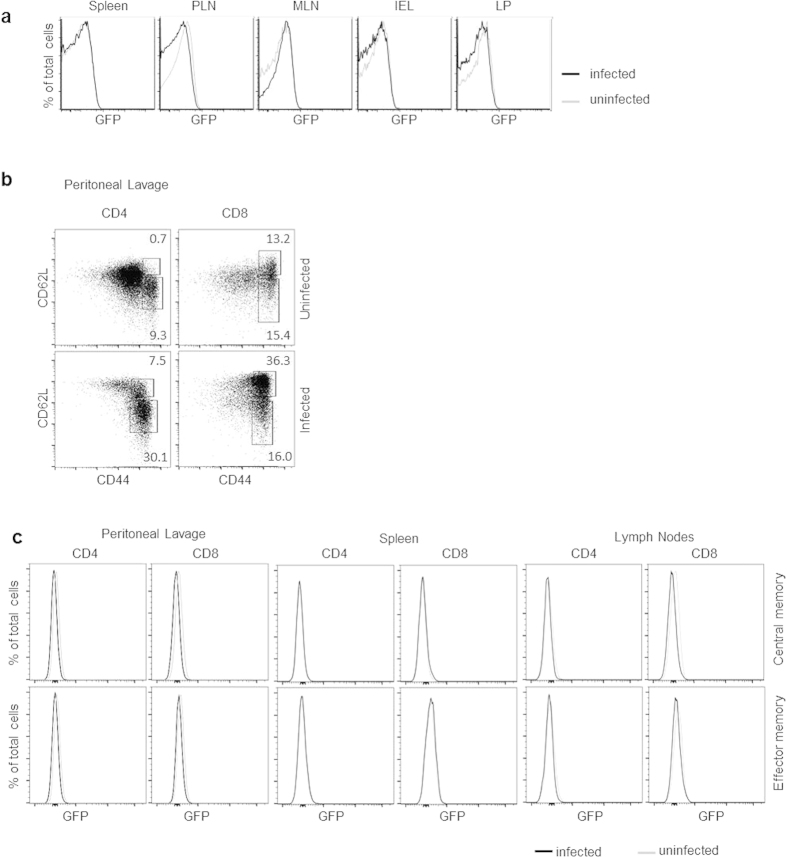
PLZF is not induced in effector or memory T cells following infection with *Listeria monocytogenes*. (**a**) Nine days after infection by 2 × 10^9^ *Listeria* by oral gavage, cells from the spleen, peripheral lymph nodes (PLN), mesenteric lymph nodes (MLN), intraepithelial lymphocytes (IEL) and lamina propria (LP) from PEG mice were harvested. Uninfected PEG mice were used as controls. Histograms show GFP expression in T cells from the indicated tissues. (**b**) PEG mice were re-challenged with *Listeria* (1 × 10^4^, i.p.) two months after primary infection. Three days later, effector memory (CD44^+^CD62L^lo^) and central memory cells (CD44^+^CD62L^hi^) T cells were analyzed. Cells were gated on the DAPI^−^CD45.2^+^CD1d-tetramer^-^ γδTCR^−^ population. (**c**) Effector and central memory T cells from peritoneal lavage, spleen, and lymph nodes generated in (**b**) were analyzed for GFP expression by FACS. Representative FACS plots from 1 of 3 experiments are shown.

Our data clearly show that TCR mediated signals are not sufficient to induce PLZF in T cells and in developing thymocytes. We next asked if heritable repression of PLZF was maintained during the inflammatory conditions induced during bacterial infection. To test this, PEG and WT mice were infected with *Listeria monocytogenes* via oral gavage. Oral gavage was utilized rather than intravenous injection since it is the natural route of infection. Nine days after infection, we examined effector T cells from each of the tissues. Results showed no PLZF expression in the effector T cells (Fig. 6a).

We extended our analysis of the infected mice to directly analyze the possibility that PLZF expression is induced in memory T cells. Previously infected mice were re-challenged with *Listeria* by intraperitoneal injection two months after the initial infection. This secondary infection resulted in the generation of both effector and central memory CD4^+^ and CD8^+^ T cells demonstrating that the infection induced an effective immune response (Fig. 6b). PLZF expression, based on upregulation of GFP, was determined for effector memory (CD44^+^CD62L^lo^) and central memory cells (CD44^+^CD62L^hi^). For all examined populations, there was no detectable upregulation of PLZF (Fig. 6c). Therefore, suppression of PLZF expression in non-innate T cells is stably maintained even under inflammatory conditions.

## Discussion

Plasticity of T cells, the conversion of one effector subset into another, is considered to be important aspect of the immune response. At its basis, is the apparent ability of T cells to downregulate key transcription factors in lieu of the upregulation of others. For example, it appears that the key regulatory T cell transcription factor FoxP3, can be transiently induced in CD4 T cells, endowing effector T cells with suppressor qualities. Furthermore, FoxP3 expressing cells have been shown to differentiate into Bcl6 expressing T follicular helper (Tfh) cells or RORγt expressing IL-17 producing Th17 cells^38^. CD4 T cells have been shown to downregulate ThPOK in the gut, resulting in upregulation of CD8^39^. Therefore, modulation of key transcription factors in differentiated cell types may be an essential feature for a fully competent immune system.

Among T cells, the expression of the transcription factor, PLZF, appears to be restricted to αβ NKT cells^3^,^4^ and the so-called γδ NKT cells (Vγ1.1 Vδ6.3 T cells)^6^,^40^,^41^. αβ NKT cells express PLZF from their earliest stages of development and, as far as is known, throughout the lifespan of each cell^3^. Expression is initiated in the thymus, likely coincident with positive selection at the double positive stage of development, although expression prior to this developmental checkpoint has not been ruled out. In the absence of PLZF, the potent, rapid innate-like response of NKT cells is lost. Indeed, in many ways, PLZF deficient NKT cells resemble conventional, naïve CD4 T cells^3^,^4^.

Ectopic expression of PLZF in conventional CD4 and CD8 T cells results in the spontaneous acquisition of effector functions that requires neither agonist TCR signals^17^ nor signals necessary for the development of NKT cells^15^. Tightly controlled expression of PLZF, therefore, appears to be essential both for maintaining the functionality of innate T cells and for preventing unpredictable immune responses in conventional T cells. Recent reports, however, have indicated that PLZF can be induced simply by strong TCR mediated signaling^18^,^23^,^24^. This concept has rapidly been accepted by others^19^,^25^,^32^,^42^,^43^,^44^.

Here, we have used sensitive approaches, including molecular fate mapping, to definitively show in several different experimental systems, that PLZF is stably suppressed in non-innate T cells. The failure to induce PLZF in conventional T cells is supported by data mined from genome wide mapping of studies of histone modifications^45^. H3K4me3 and H3K27me3 modifications are associated with regions of the chromatin that are active and inactive in terms of the likelihood of gene expression, respectively^46^,^47^. Evaluation of these data showed that in naïve T cells, *zbtb16* is prominently decorated with the inactive H3K27me3 in contrast to little detectable H3K4me3 active chromatin marks (Supplementary Fig. 4), suggesting that failure to express PLZF is enforced by this epigenetic signature. This conclusion is also drawn from an analysis of the same histone markers from Th17 T cells, Th2 T cells and Th1 T cells. These chromatin modifications imply that expression of PLZF is actively repressed in naïve T cells and, since these effector cells represent the direct progeny of the naïve cells, the suppression of expression is epigenetically maintained following activation and differentiation into effector T cell subsets.

We have previously shown that, unexpectedly, CD4 T cells can be positively selected by MHC class II expressed by other thymocytes^48^. Interestingly, approximately half of the cells selected via this pathway are induced to express PLZF^26^. One possible explanation was that the PLZF expressing cells received a stronger TCR mediated signal during selection. However, we have also shown T cells expressing a transgene encoded TCR specific for thymocyte expressed MHCII are also only ~50% positive for PLZF expression^26^. Therefore, thymocytes bearing the identical TCR, which presumably delivers a similar strength of signal to all cells, results in divergent PLZF expression. It has also been shown that sustained TCR signaling is not necessary for maintaining PLZF since ablation of the TCR in mature NKT cells has no effect on expression^49^. Conversely, molecular replacement of TCRs expressed by conventional T cells with the invariant NKT TCR does not induce PLZF expression^49^. It is also interesting to note that Id3, which is argued to be a key downstream effector molecule of strong TCR signals^50^, is expressed at very low levels in stage 0 NKT cells^19^, suggesting that strong signaling early in NKT cell development may not be critical for development. Indeed, GFP expression at stage 0 of NKT cell development from a Nur77 transgene that is reported to indicate the strength of TCR signaling is similar to what is seen for conventional CD4 T cells (Supplemental Fig. 5a,b).

Collectively, our data show that PLZF is inactivated early during thymocyte development and this inactivation is stably and heritably maintained in all T cells other than innate T cells. Therefore, unlike nearly all other T cell expressed lineage specifying transcription factors, PLZF expression is extraordinarily specific. The eventual understanding of the signals that initiate expression of this transcription factor will likely be the key to understanding the development of innate T lymphocytes^51^. Furthermore, it will be important to determine if other members of the BTB-ZF transcription factor family are also resistant to expression mediated by receptor signaling and cellular activation.

## Methods

### Mice

*Rosa26*-tdTomato (R26T), containing a loxp-flanked STOP cassette that prevents transcription of the tdTomato gene, Nur77^GFP^, which carry a BAC transgene with GFP replacing the Nur77 gene, DO11.10, with carry transgenes encoding the DO11 TCR and C57BL/6J mice were purchased from The Jackson Laboratory (Bar Harbor, ME). The PEG reporter mice were generated in our lab with a modified bacterial artificial chromosome transgene expressing eGFP under the control of PLZF regulatory elements. The pLD53 SC-AB vector system (provided by Dr. E. Pamer, MSKCC, New York, NY) was used for the modification. The purified bacterial artificial chromosome transgene was microinjected into fertilized C57BL/6 eggs by MSKCC’s Mouse Genetics Core Facility. GFP expression faithfully reproduced PLZF expression in all three founder lines. One was selected for further experiments^26^. PCre mice were generated with similar methodology with a bacterial artificial chromosome transgene expressing Cre recombinase under the control of PLZF promoter. All mouse strains were bred and maintained in the animal facility of the Child Health Institute of New Jersey. All experimental protocols and procedures were approved by the Institutional Animal Care and Use Committee of the Child Health Institute of New Jersey. Animal Care and experimental procedures were carried out in accordance with the guidelines of the Institutional Animal Care and Use Committee of the Child Health Institute of New Jersey and the National Institutes of Health Guide for the Care and Use of Laboratory Animals.

### Flow Cytometry

Cells were harvested from the tissues and preincubated with the anti-FcγR mAb and normal mouse serum to block nonspecific antibody binding for 15 min at 4 °C. Surface staining was performed in FACS buffer (PBS with 1% heat inactivated FBS) for 20 min at 4 °C using the indicated surface Abs. Dead cells were excluded by DAPI staining and doublet events were eliminated by comparing the FSC-W to the SSC-W. Events were acquired on a LSRII cytometer (BD Biosciences, San Jose, CA), and the data were analyzed with the FlowJo software (TreeStar, Ashland, OR). PLZF intracellular staining was performed using the Foxp3 eBiosciences (San Diego, CA) kit.

### Abs

PBS57-loaded CD1d-tetramer was provided by the National Institutes of Health tetramer core facility. The following Ab clones were used: anti-CD3 (500A2), anti-CD4 (RM45), anti-CD8 (53-6.72), anti-CD24 (M1/69), anti-CD25 (PC61), anti-CD44 (IM7), anti-CD45.2 (104), anti-CD69 (H1.2F3), anti-PLZF (Mags.21F7), purified anti-CD3 (2C11), purified anti-CD28 (37N), purified anti-Ly108 (13G3-19D). Abs were used with different fluorochrome conjugations: FITC- PE-, PerCP-Cy5.5-, PE-Cy7-, APC-, APC-Cy7-, AF700-, and Pacific Blue.

### *In Vitro* Stimulation and Cell Culture

1 × 10^5^ sorted CD4 or CD8 conventional T cells (purity >98%) were incubated in 200 ul complete RPMI 1640 plus 5% FBS, 1% Pen/Strep, 2 mM L-glutamine, and 0.05 mM 2-ME in 96-well round bottom plates coated with or without plate-bound anti-CD3 (2C11, 5 μg/ml) and soluble anti-CD28 (37N, 5 μg/ml) for 1, 2 or 3 days. Preselection DP (PS-DP) thymocytes were isolated by negative selection using CD3, CD25 and CD44 Abs. 1 × 10^6^ sorted PS-DP thymocytes (purity >98%) were incubated in media in 96-well round bottom plates coated with or without plate-bound anti-CD3 (2C11, 5 μg/ml), soluble anti-CD28 (37N, 5 μg/ml) and anti-Ly108 (13G3-19D; eBiosciences, 5 μg/ml) for 18, 24 or 48 hours. Cells were harvested at each time point and analyzed by FACS. For PLZF intracellular staining, cells were stained with the Live/Dead Fixable Dead Cell stain kit (Invitrogen). Then the cells were fixed and stained with the monoclonal anti-PLZF antibody.

### Adoptive T Cell Transfer

CD45.2^+^tdTomato^−^ T cells were sorted by FACS from the thymus or spleen of PCre × R26T mice. 2 × 10^6^ sorted cells (purity >99%) were i.p. injected into CD45.1^+^ B6.SJL recipient mice, followed by i.p. injection of purified anti-CD3 (2C11, 50 μg/mouse). Two weeks later, cells were harvested from the spleen, lymph nodes and liver of recipient mice. CD45.2^+^ donor cells were analyzed by FACS.

### Listeria infection

Two-month old PEG and WT mice were infected with 2 × 10^9^ *Listeria monocytogenes* by oral gavage. Nine days later, the mice were sacrificed. Spleen, peripheral lymph nodes, mesenteric lymph nodes, intraepithelial lymphocytes and lamina propria were harvested for the study of PLZF expression in effector T cells. To study PLZF expression in memory T cells, the mice were i.p. injected with 1 × 10^4^ *Listeria* two months after the oral gavage. Three days after injection, the mice were sacrificed. Spleen, peripheral lymph nodes, mesenteric lymph nodes, peritoneal lavage, intraepithelial lymphocytes and lamina propria were harvested and analyzed by FACS.

### Statistical Analysis

Statistical analysis was performed using GraphPad Prism (La Jolla, CA) software. All samples were analyzed using unpaired, two-tailed Student *t* test.

## Additional Information

**How to cite this article**: Zhang, S. *et al. Zbtb16* (PLZF) is stably suppressed and not inducible in non-innate T cells via T cell receptor-mediated signaling. *Sci. Rep.* **5**, 12113; doi: 10.1038/srep12113 (2015).

## Supplementary Material

Supplementary Information
