# Supplementary material for: Zbtb16 (PLZF) is stably suppressed and not inducible in non-innate T cells via T cell receptor-mediated signaling
Source: Sci Rep. 2015 Jul 16;5:12113. doi: 10.1038/srep12113 (PMC4503983; doi:10.1038/srep12113)
Supplement: Supplementary Information [file srep12113-s1.pdf]

Supplementary Information

Zbtb16 (PLZF) is stably suppressed and not inducible in non-innate T cells via T cell receptor-mediated signaling

Sai Zhang[1,4], Amale Laouar[2,4], Lisa K. Denzin[1,3,4] and Derek B. Sant'Angelo[1,3,4]

[1]Graduate School of Biomedical Sciences
[2]Department of Surgery
[3]Department of Pediatrics
[4]Child Health Institute of New Jersey
Rutgers Robert Wood Johnson Medical School
New Brunswick, NJ 08901, USA

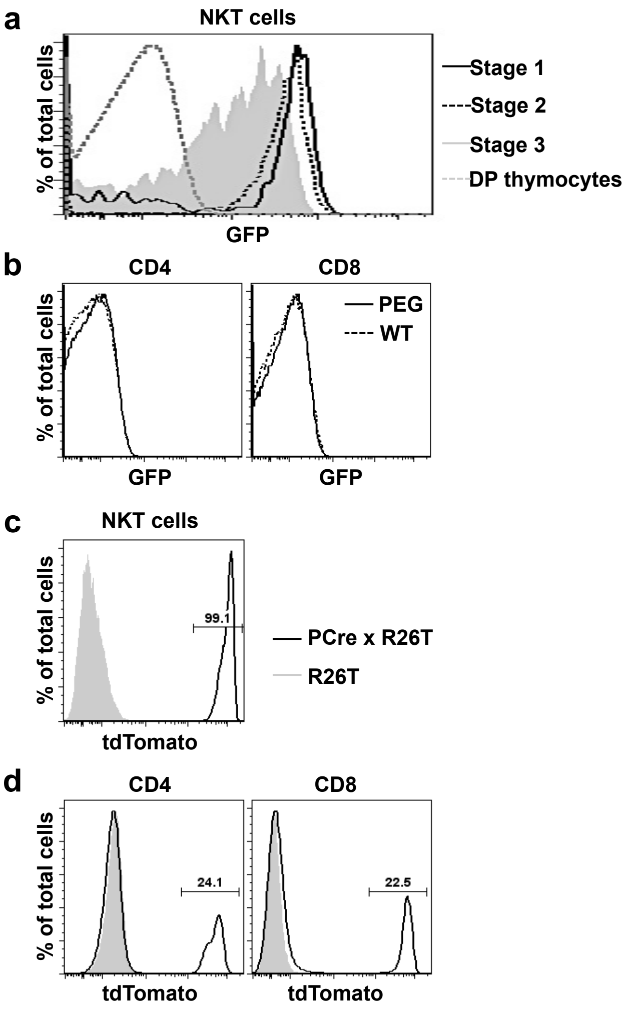

Supplementary Figure 1 | Two reporter systems used to monitor PLZF expression. (**a**) GFP expression mimics PLZF expression in NKT cells at different stages of development. (**b**) GFP expression is not detected in CD4SP and CD8SP thymocytes. (**c**) Nearly all NKT cells from PCre x R26T mice express TdTomato, as anticipated (**d**). Similar to a previous report, 20-30% of CD4SP and CD8SP thymocytes from R26T and PCre x R26T mice express TdTomato.

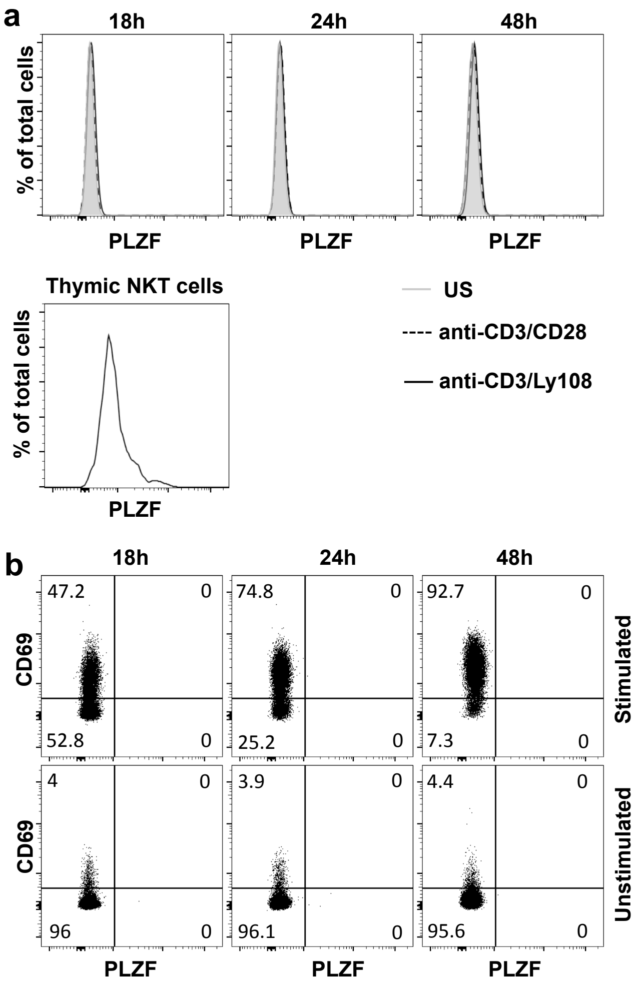

Supplementary Figure 2 | Intracelullar PLZF staining of PS-DP thymocytes in WT mice. (**a**) PS-DP thymocytes were sorted from WT mice, stimulated with anti-CD3/anti-CD28 or anti-Ly108 for up to 48 hours, then analyzed by FACS. PLZF was stained intracellularly with a monoclonal anti-PLZF antibody. Thymic NKT cells (bottom) were stained with the same antibodies as a positive control. (**b**) Sorted cells were also stained for surface expression of CD69 after stimulation. Numbers in dot plots show the percentage of events in each quadrant. Representative FACS plots from 1 of 3 independent experiments are shown.

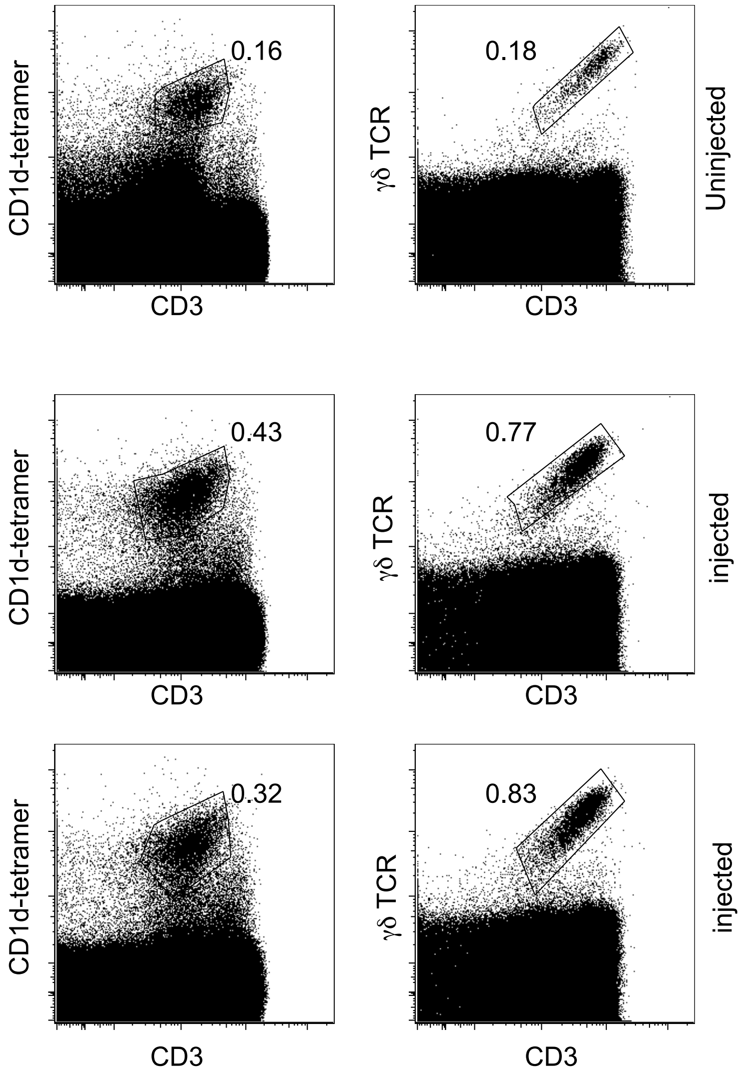

Supplementary Figure 3 | Increase of NKT cells and γδ T cells following injection of anti-CD3. PEG mice were intravenously injected with 50 ugs of anti-CD3. 30 hours later, the mice were euthanized and thymocytes were analyzed. DAPI negative (live), single events were analyzed as shown. Numbers indicate the percentage of cells within the gate. Results from two mice that were injected with anti-CD3 are shown.

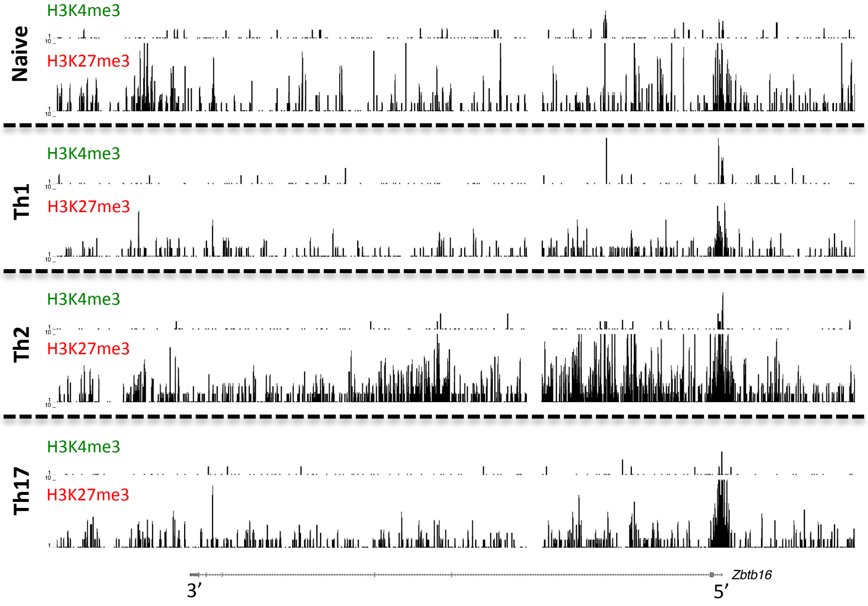

Supplementary Figure 4 | Histone modifications suggest *zbtb16* gene transcription is actively suppressed in non-innate T cells. The distribution of activating H3K4me3 (green) and inactivating H3K27me3 (red) modifications along the *zbtb16* genomic DNA is shown. The cell type examined (naïve, Th1, Th2 and Th17 cells) is indicated to the right. The direction of transcription of the *zbtb16* gene is noted at the bottom of the figure. Database was originally published as a resource in Wei et al "Global Mapping of H3K4me3 and H3K27me3 Reveals Specificity and Plasticity in Lineage Fate Determination of Differentiating CD4^{+} T Cells" *Immunity* **30**,155–167, 2009.

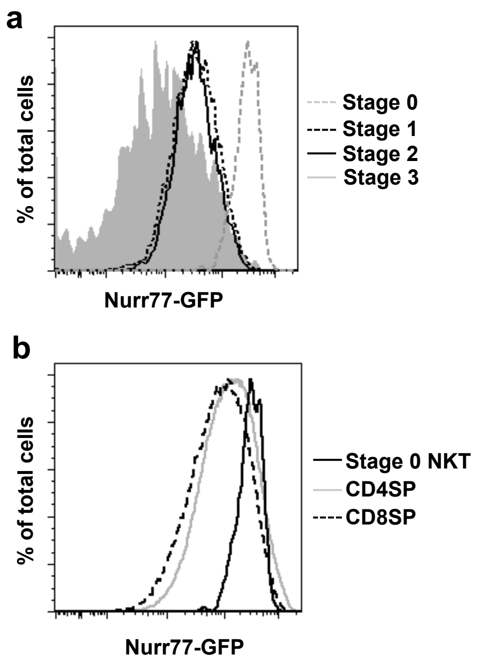

Supplementary Figure 5 | Nur77-GFP expression in NKT cells overlaps with conventional T cells. (**a**) CD1d-tetramer-binding NKT cells from Nur77^{GFP} mice were enriched from adult thymus using magnetic beads. GFP level in NKT cells at different stages of development are shown. (**b**) GFP levels of stage 0 NKT cells compared with conventional CD4SP and CD8SP T cells of Nur77^{GFP} mice are shown.
